# Practical Notes and Formulæ

**Published:** 1879-11-20

**Authors:** 


### PRACTICAL NOTES AND FORMULÆ.

**Fever in a Child.**—When the child is feverish, tinct. aconite in dose proportionate to its age must be given. If there is vascular irritation of the brain, characterized by bright eyes and flushed face, give tinct. gelsemium in connection with aconite.

| | | | |
|---|---|---|---|
| ℞ | Tinct. aconite | 5 drops. | |
| | Tinct. gelsemium (green root) | 10 drops. | |
| | Vini ipecac | 20 drops. | |
| | Aqua | 4 ounces. | M. |

Dose, for a child six to nine months old, give one tablespoonful every hour until the symptoms are relieved.

When the vomiting is persistent the following may be given :

| | | | |
|---|---|---|---|
| ℞ | Vini ipecac | ½ drachm. | |
| | Bismuth sub. nit | 20 grains. | |
| | Sodii sulphis | 10 grains. | |
| | Aqua | 4 ounces. | M. |

Dose, one teaspoonful every hour until the stomach is quiet.

Syrup of lime, ten drops, with a wine glass of milk—half of this every two hours, when the vomiting depends upon gastric irritation. —*Brief.*

**Anteflexion of the Uterus.**—The patient was a servant girl in New York Hospital, twenty-seven years of age, with a history of menstrual irregularities extending through a period of seven years. Accompanying the flow there had been occasional supra-pubic pain. Six months before her admission to the hospital this pain had become constant and she had been compelled to give up all work. She was obliged to pass her water twenty or thirty times a day. The urine was examined, and found to be entirely normal in all respects.

Vaginal examination showed the uterus to be a little lower than natural; the finger encountering the fundus in the anterior cul-de-sac. Together with this anteflexion there was some catarrh of the bladder, while the woman was anæmic and hysterical, and suffered greatly from constipation.

It was concluded that the first thing to do was to build up the woman's general health. Rest in bed was enjoined; thrice a day she took gr. iv. of the ammonio-citrate of iron with gentian, and the following prescription was employed, viz :

| | | | |
|---|---|---|---|
| ℞ | Magnesii sulphat | ʒ vj. | |
| | Acid. sulph. dil | ʒ ij. | |
| | Ferri sulph | gr. xij. | |
| | Quin. sulph | gr. xij. | |
| | Syrup. zingiberis | f. ℥ j. | |
| | Aquæ q. s. ad | f. ℥ vj. | M. |

Dose, a tablespoonful in ice-water thrice daily.

To cure the anteflexion, instead of introducing a pessary, it was determined to persuade the woman to teach her bladder to hold gradually more and more urine. It was reasoned that when the bladder could hold twelve ounces, the anteflexion would be largely reduced.—*Med. Record.*

**Post Nasal Catarrh.**—Dr. Mays, in *Brief*, says: A solution of salt and water, a teaspoonful to a pint of tepid water, passed through the nasal passages once a day, has cured many a case of chronic catarrh.

The best course of treatment is to cleanse the diseased surfaces with the post-nasal syringe, throwing up behind the palate a warm solution of bicarbonate of soda, which dissolves the mucus and prepares the way for the various astringents and detergents which are then used in the form of spray. There is a variety of these, such as sulphate of zinc, chlorate of potash, perchloride of iron, permanganate of potassa, tannin, carbolic acid, glycerine in various combinations as may be found best suited to the case.

These applications should be made twice a day, and continued until the patient is relieved. They should all be used warm, as any cold solution would be attended with danger. Very weak solutions are to be used, as the diseased surfaces are very sensitive, and any solution strong enough to give pain will do more harm than good. The patient generally gets relief from, and is willing to continue the treatment as long as necessary. But there are some old cases in scrofulous constitutions, where all we can do is to afford very great relief, but cannot effect a perfect cure. I usually treat these cases three weeks at a time, and advise them to come again after a few months and undergo another course of the same length of time. The patient should be very careful to avoid exposure to cold and damp while he is being treated. He also uses a gargle in some cases, as follows:

℞. Hydrochlorate ammonia............. 2 drachms.
Pulv. alum...............................30 grains.
Aqua.................................... 6 ounces.
Several times a day.

**Ophthalmia Neonatorum.**—A solution of nitrate of silver (three-quarters of a grain to the ounce) is injected under the lids twice a day.

For the lids themselves the following is usually employed:

℞ Sodæ boratis .................................... gr. xij.

Zinci sulphatis.................................... gr. j.

Aquæ camph...................................... f. ʒ j.

Aquæ destill....................................... f. ℥ j. M.

To be applied to the lids two or three times a day.—*New York Med. Record.*

**Liquid Glue.**—Take of best white glue, or better, of best French transparent gelatine, 8 parts. Cut it fine, cover it with distilled water, and soak it until soft; then pour off any excess of water. Melt it on a water-bath, and add to it one part of glacial acetic acid. When thoroughly mixed, bottle it.—*New Remedies.*

**Treatment of Barber's Itch.**—Dr. Brame recommends the following treatment: Shave off the hair or cut them very short; then apply once or twice a week an ointment composed of:

| | | |
|---|---|---|
| Prepared chalk | 10 | parts. |
| Coal-tar | 1 to 4 | " |
| Glycerin | 5 | " |
| Simple cerate | 50 | " |

or the following:

| | | |
|---|---|---|
| Prepared chalk | 8 | parts. |
| Coal-tar | 1 to 4 | " |
| Linseed oil | 20 | " |

—*La Ruche Pharmaceutique.*

**Popp's Anatherine Dentifrice.**—

| | | |
|---|---|---|
| ℞ | Cloves | 4 ʒ. |
| | Red saunders | 4 ʒ. |
| | Guaiac wood | 160 gr. |
| | Cinnamon | 160 gr. |
| | Myrrh | 380 gr. |
| | Oil of cloves | 10 gtt. |
| | Oil of cassia | 10 gtt. |
| | Alcohol | 3 fl. ℥. |
| | Rose water | 26 fl. ℥. |

—*Hager's Manuale.*

**Lip Salve.**—The following formulæ are recommended:

| | | | |
|---|---|---|---|
| ℞ | Spermacetti | 40 | parts. |
| | Lard, perfectly pure and fresh | 80 | " |
| | White wax | 20 | " |
| | Oil of sweet almonds | 5 to 10 | " |

according to the season of the year, are melted together, the mixture colored with a sufficient quantity of alkanet, by digesting the root with the melted mass, and the latter then suitably perfumed, for instance, with:

| | | |
|---|---|---|
| Oil bergamot | 2 | parts. |
| Oil orange | 3 | " |

The mass is then poured out into moulds. It is customary to pour it into tin-tubes, from which it is removed when cold, and then covered with tin-foil.—*New Remedies.*

**Glycerole of Thymol.**—The formula is:

| | | | |
|---|---|---|---|
| ℞ | Thymol | 20 | grs. |
| | Glycerin | 1 | ounce. |
| | Rectified spirit | 1 | " |
| | Distilled water | 16 | " |

Useful in pityriasis, and, when diluted, as an effective antiseptic mouth-wash.

It is said that thymol has the property of immediately removing the smell of tobacco.—*New Remedies.*

**Remedial Management of Acute Testitis.**—By Z. C. McElroy, M. D. (Cincinnati *Lancet and Clinic*, October 4, 1879.) The management of this painful disease, by which the doctor claims better results than commonly have been attained, consists in hypodermic injection of morphiæ sulph., in ¼ gr. doses, into the cellular tissue of the scrotum, and internally:

℞ Calomel........................gr. iij.
Ipecac pulv.......................gr. x.
M. Sig.—Take at once.

To have saline in the morning and follow with smaller doses of calomel and ipecac every six hours. Repeat hypodermics of morphiæ, p. r. n. Apply locally:

℞ Hydrarg ammoniati..................ʒ j.
Cerati simp...............................℥ j.
M. ft. ungt.
Sig.—Apply as directed.

Case dismissed on the fourth day.

[We have obtained equally as good and rapid results by the use of saline cathartic and an application of belladonna extract, made soft with water, and smeared over the scrotum.—ED.]—*Detroit Lancet*.

**Ulcers and Abrasions.**—Dr. Culver, in *Brief*, states: As a local application in ulcers and abrasions I can find nothing to equal the following:

℞. Glycerrhina.............. } quantiiies sufficient.

Rhei pulv................ }

Mix. Fiat; paste.

Put this on the ulcer in sufficient amount to more than cover the ulcer or abrasion and spread it moderately thick with a knife. It hermetically seals the parts to be treated and I let it stay on as a permanent dressing, and it is surprising how remarkably quick the healing process takes place.

**Russian Spirit.**—There is a liniment known under this name, which is used for rheumatism. The formula is:

| | | |
|---|---|---|
| ℞ Olei sinapis destillati | 12 | parts. |
| Olei thereb | 75 | " |
| Camph | 75 | " |
| Liquor am. fort | 75 | " |
| Tinct. capsici | 75 | " |
| Alcoholis fort | 1000 | " |

A MAN was recently convicted of a petty theft before a police court. He had once been a prominent physician, and dated his downward course from the time that he cheated the publisher of his medical journal out of the subscription price. After that he said that every piece of rascality came easy to him. The moral here needs not be pointed out, and we shudder for the future of some.—*Michigan Medical News*.

**Ptyalism.**—

| | | |
|---|---|---|
| ℞ Hypophosphite of soda | 2 | drachms. |
| Hypophosphite of lime | 2 | drachms. |
| Aqua | 4 | ounces. M |

Dose, tablespoonful every four hours.—Dr. Burner, in *Brief*.

